# Integration of HPV6 and Downregulation of AKR1C3 Expression Mark Malignant Transformation in a Patient with Juvenile-Onset Laryngeal Papillomatosis

**DOI:** 10.1371/journal.pone.0057207

**Published:** 2013-02-20

**Authors:** Christian Ulrich Huebbers, Simon Florian Preuss, Jutta Kolligs, Julia Vent, Markus Stenner, Ulrike Wieland, Steffi Silling, Uta Drebber, Ernst-Jan M. Speel, Jens Peter Klussmann

**Affiliations:** 1 Jean-Uhrmacher-Institute for Otorhinolaryngological Research, University of Cologne, Cologne, Germany; 2 Department of Otorhinolaryngology, Head and Neck Surgery, University Hospital of Cologne, Cologne, Germany; 3 Institute of Virology, National Reference Centre for Papilloma- and Polyomaviruses, University Hospital of Cologne, Cologne, Germany; 4 Institute for Pathology, University Hospital of Cologne, Cologne, Germany; 5 Department of Pathology, GROW-School for Oncology and Developmental Biology, Maastricht University Medical Center, Maastricht, The Netherlands; 6 Department of Otorhinolaryngology, Head and Neck Surgery, University Hospital of Giessen, Giessen, Germany; IPO, Inst Port Oncology, Portugal

## Abstract

Juvenile-onset recurrent respiratory papillomatosis (RRP) is associated with low risk human papillomavirus (HPV) types 6 and 11. Malignant transformation has been reported solely for HPV11-associated RRP in 2–4% of all RRP-cases, but not for HPV6. The molecular mechanisms in the carcinogenesis of low risk HPV-associated cancers are to date unknown. We report of a female patient, who presented with a laryngeal carcinoma at the age of 24 years. She had a history of juvenile-onset RRP with an onset at the age of three and subsequently several hundred surgical interventions due to multiple recurrences of RRP. Polymerase chain reaction (PCR) or bead-based hybridization followed by direct sequencing identified HPV6 in tissue sections of previous papilloma and the carcinoma. P16^INK4A^, p53 and pRb immunostainings were negative in all lesions. HPV6 specific fluorescence in situ hybridization (FISH) revealed nuclear staining suggesting episomal virus in the papilloma and a single integration site in the carcinoma. Integration-specific amplification of papillomavirus oncogene transcripts PCR (APOT-PCR) showed integration in the aldo-keto reductase 1C3 gene (AKR1C3) on chromosome 10p15.1. ArrayCGH detected loss of the other gene copy as part of a deletion at 10p14-p15.2. Western blot analysis and immunohistochemistry of the protein AKR1C3 showed a marked reduction of its expression in the carcinoma. In conclusion, we identified a novel molecular mechanism underlying a first case of HPV6-associated laryngeal carcinoma in juvenile-onset RRP, i.e. that HPV6 integration in the AKR1C3 gene resulted in loss of its expression. Alterations of AKR1C gene expression have previously been implicated in the tumorigenesis of other (HPV-related) malignancies.

## Introduction

Recurrent respiratory papillomatosis (RRP) is a benign neoplasm of the larynx, predominantly induced by low risk HPV types 6 and 11 [1], [2]. RRP may occur at any site in the airway tract, although the larynx is the preferred location [3]. The viral etiology of RRP was first suggested by Ullmann in 1923 [4] and has been confirmed by HPV6/11-specific in situ hybridization and polymerase chain reaction (PCR) [5]. In adult-onset RRP, both HPV types 6 and 11 can be found, whereas in juvenile-onset RRP, mainly HPV11 is detected [6]–[9]. It is known that RRP has the potential to undergo malignant transformation. The reported incidence of carcinoma developing in RRP patients varies from 0% to 22% [3], [10]. In our own series of patients treated at the University of Cologne, the incidence of airway carcinoma was 4.6% [11].

To date, it is unclear how a low risk HPV infection may lead to malignancy. Several extrinsic factors could primarily be responsible for some of the malignant transformations. Particularly, smoking and previous radiation therapy may independently induce malignant transformation, or facilitate viral integration in DNA damaged areas of the genome. Alternatively in cases without such risk factors, the virus may incidentally integrate into genes that are actively transcribed [12]–[15], as has been suggested to occur in high risk HPV-related anogenital and oropharyngeal cancers [16]–[18].

Here we studied for the first time consequences of a rare case of low risk HPV6-integration in juvenile onset RRP in relation to malignant transformation.

## Materials and Methods

### Patient and tumor materials

A 24-year old female patient that never smoked presented with an advanced laryngeal carcinoma. The patient showed a long history of RRP. At the age of three, she had undergone a first microlaryngoscopy with removal of laryngeal masses, which were diagnosed as papillomatosis in the histopathologic investigation. Several hundred revision surgeries had been necessary to control recurrences of the disease. In 2008, a further microlaryngoscopy and histopathologic examination revealed a malignant laryngeal squamous cell carcinoma of stage IV. Total laryngectomy with bilateral neck dissection was performed. Adjuvant chemoradiation with 5-FU and carboplatin to a total dose of 70 Gy was administered postoperatively. However, the patient developed inoperable paratracheal recurrence of the primary tumor and eventually died 21 months after the diagnosis of recurrence. The patient had never been treated with Cidofovir.

We analyzed formalin-fixed, paraffin-embedded tissue that had been archived at the Institute of Pathology at the University of Cologne Medical Center from previous resections (1985 and 1989) of the laryngeal papilloma, as well as fresh frozen tumor tissue from the laryngeal carcinoma and lymph node metastasis (2008). Furthermore, we analyzed 9 additional, randomly chosen fresh frozen tissue samples from our tumor tissue archive, i. e. three cases of laryngeal papilloma (two HPV6- and one HPV11-positive), three HPV16-associated oropharyngeal squamous cell carcinomas (OSCC) and three HPV-negative oropharyngeal squamous cell carcinomas. These samples served as controls for immunohistochemical stainings and Western blotting.

### Ethics Statement

The ethics committee of the University of Cologne medical faculty approved this study. Written, informed consent had been obtained from the hereby reported patient and all other patients whose material was used for control. All control samples were anonymized prior to analysis.

### Detection of HPV-DNA by PCR

Genomic DNA was extracted from five 10 µm-thick formaldehyde-fixed, paraffin-embedded tissue sections and of snap frozen tissue sections, respectively, using the Gentra puregene tissue kit with proteinase K treatment (Qiagen, Hilden, Germany) according to the manufacturer's protocol.

Paraffin was removed by graded xylol and ethanol baths before extraction of the DNA. Both a general primer GP5+/6+ PCR (150 base pairs [bp] product) and a nested PCR with degenerate primers A5/A10 (527 bp product) and A6/A8 (268 bp product) for HPV detection were applied [19]. Five µl of these PCR products were separated on a 1.5% agarose gel and visualized by ethidium bromide staining. Direct sequence analysis of purified PCR products (QIAquick PCR purification kit, Qiagen, Hilden, Germany) was carried out with an ABI Prism 377 DNA sequencer using the Taq FS Big-Dye-Terminator cycle sequencing method (PE Applied Biosystems, Weiterstadt, Germany). Human papillomavirus typing was performed by comparison of the obtained sequences with database entries using NCBI Blast search. Additionally, HPV-typing was performed by bead-based multiplex hybridization of A6/A8 PCR products with 38 type-specific probes as previously described [20]. HPV-types covered by the assay were HPV6, 11, 16, 18, 26, 30, 31, 33–35, 39, 40, 42–45, 51–59, 61, 66–68, 70–73, 81–84, 89 [20], [21].

β-globin gene PCR served as a positive control for sufficient DNA of adequate quality, and to show that samples were free of PCR inhibitory substances (268 bp PCO4/GH20 PCR product) [22].

### Immunohistochemical staining

The following primary antibodies were used: monoclonal mouse anti-human p16^INK4A^ antibody, clone G175-405 (BD Biosciences, Heidelberg, Germany); monoclonal mouse anti-human AKR1C3 antibody, clone NP6-G6.A6 (does not cross react with AKR1C1, AKR1C2, and AKR1C4) (Sigma-Aldrich, Munich, Germany), monoclonal mouse anti-human p53 antibody, clone DO-7 (BioLogo, Kronshagen, Germany), and monoclonal mouse anti-human pRb antibody, clone J146-35 (BD Biosciences, Heidelberg, Germany).

Immunohistochemical staining on 5 µm-thick formaldehyde-fixed, paraffin-embedded tissue sections and cryostat sections, and slide evaluation was performed as previously described [23], [24]. Primary antibodies were detected using the avidin-biotinylated peroxidase complex (ABC) procedure (Vectastain-Elite-ABC kit; Vector Laboratories, Burlingame, USA) and peroxidase activity was visualized using diaminobenzidine/H_2_O_2_ (BD Biosciences, Heidelberg, Germany). Sections were counterstained with hematoxylin and mounted in Histofluid (Marienfeld, Lauda-Koenigshofen, Germany). Each analysis included negative and positive controls. Analysis was performed by two independent researchers (CUH and SFP) and consensus was achieved. Strong nuclear and cytoplasmic staining was considered positive for AKR1C3 expression.

### Detection of HPV6 DNA by FISH

Fluorescence in situ hybridization (FISH) for the detection of HPV6 on 5 µm thick formaldehyde-fixed, paraffin-embedded tissue sections, and evaluation of nuclear FISH signals was performed as previously described [25]–[27]. In brief, sections were deparaffinized, treated with 85% Formic acid/0.3% H_2_0_2_ and dehydrated in an ethanol series containing 0.01 M HCl (acidic dehydration). The digoxigenin-labeled HPV6 probe (Panpath, Budel, The Netherlands) was applied under a coverslip at a concentration of 1 ng/µl in 50% formamide, 2× SSC pH 7.0, 10% dextran sulphate, and a 50× excess of carrier DNA (salmon sperm DNA) followed by hybridization over night. Detection was carried out with peroxidase-conjugated sheep anti-digoxigenin Fab fragments (Roche, Basel, Switzerland; 1∶100 diluted in 4×SSC containing 5% nonfat dry milk), followed by a tyramide signal amplification (TSA) reaction using rhodamine-labeled tyramide 25–27. After dehydration in an ascending ethanol series, slides were mounted in Vectashield (Vector Laboratories, Burlingame, USA) containing 0.2 µg/ml 4,6-diamidino-2-phenyl indole (DAPI; Sigma, Germany). Slides were evaluated under a Leica DM-RE fluorescence microscope equipped with DAPI and rhodamine filters and images were recorded with the Metasystems Image Pro System (black and white CCD camera; Sandhausen, Germany).

### RNA extraction and reverse transcription

Total RNA was extracted from five 10 µm-thick snap frozen tissue sections using the RNeasy mini kit (Qiagen) according to the manufacturer's instructions, which included DNase treatment. RNA concentration and quality were determined by RNA standard sense chips on a BioRad Experion system (BioRad, Munich, Germany). 500 ng RNA was reverse transcribed in 20 µl using oligo-dT primer (25 µM), dNTPs (10 mM each), DTT (0.1 M), 5× RT-buffer and 40 U SuperScript reverse transcriptase (Invitrogen, Karlsruhe, Germany). Quality of generated cDNA was determined by a standard GAPDH gene PCR (forward primer 5′–AATGGAAATCCCATCACC–3′, reverse primer 5′–CAGCCTTGGCAGCGCCAG–3′; 441 bp product).

### Amplification of papillomavirus oncogene transcripts (APOT) PCR

For detection of the physical status of HPV6 (episomal and/or integrated virus), a 3′-RACE APOT assay (amplification of papillomavirus oncogene transcripts) was used which was based on Klaes et al. [28] and modified for HPV6 detection. After reverse transcription of RNA, a nested PCR with a set of newly designed 5′-Primers (1^st^ 5′-primer: 5′-GGACGGACAAGATTCACAACC-3′; 2^nd^ 5′-primer: 5′-CCTGTTGCTGTGGATGTGACAGC-3′) both located in the E7 open reading frame of HPV6 and a 3′-Frohman primer (for both nested PCR-setups) was used [29]. PCR products were separated on a 1.2% agarose gel. Candidate bands for viral integration different from episomal ones were cut out of the gel, purified (Gel extraction kit, QIAGEN) and sequenced. Sequences were compared with NCBI and UCSC database entries to determine virus-human fusion points indicating viral integration.

### TP53 Sequencing analysis

Sequence analysis of TP53 was performed by PCR amplification followed by direct sequencing based on the IACR protocol [30]. Exons 4–8 were amplified from cDNA, Exons 2, 3 and 9–11 were amplified from DNA. Sequences were compared with reference sequence NC_000017.9.

### Array-based comparative genomic hybridization (aCGH)

High-resolution oligo-nucleotide aCGH was performed using a 105 K microarray (99,000 human sequence probes) (Agilent Technologies, Boeblingen, Germany) according to the manufacturer's protocol. In brief, 1.5 µg genomic tumor and reference DNA (G1521 female DNA, Promega, Mannheim, Germany) were digested with AluI and RsaI, labeled and processed according to the manufacturer's protocol and scanned using an Agilent G2567AA Scanner. Images were extracted using Feature Extraction 9.5 software and visualized using Genomic Workbench 6.5 software (both Agilent Technologies). Aberrations were calculated by the z-score algorithm with a threshold of 2.5. Primary array CGH data have been made publicly available at EMBL-EBI (Accession No. E-MEXP-3330) for use in subsequent analysis.

### Western blotting

Total protein extracts were derived from five 10 µm-thick snap frozen tissue sections. Extracts were normalized after protein concentrations were determined by bradford assay according to the manufacturer's protocol (Roti-Nanoquant, Carl Roth, Karlsruhe, Germany) and equal amounts were resolved via SDS-PAGE (sodium dodecyl sulfate polyacrylamide gel electrophoresis). Blotting of proteins on nitrocellulose membranes was carried out according to standard protocols in a semi-dry blotter using Towbin buffer (0.025 M Tris-HCl pH 8.0; 0.192 M Glycin; 20% Methanol). After blocking with 4% non-fat milk powder in TBS-T buffer (0.1% Tween 20 in Tris-buffered saline), monoclonal anti-AKR1C3 antibody (clone NP6-G6.A6, Sigma-Aldrich, Munich, Germany) followed by secondary anti-mouse IgG peroxidase conjugated antibody (Sigma-Aldrich A 9044) was applied to the membrane. Dilution of antibodies and subsequent washing was done in TBS-T buffer. For visualization, Pierce solution (Biozym, Hessisch Oldendorf, Germany) was applied to the nitrocellulose membrane. Chemiluminescence signals were documented with an Alpha Innotech Fluorchem FC2 system (Biozym). Blots were stripped and re-probed with monoclonal beta-actin antibody (Clone AC-74, Sigma-Aldrich) as control.

## Results

Genomic DNA was extracted from paraffin-embedded laryngeal lesions of the patient from the years 1985 (papillomatosis), 1989 (papillomatosis) and 2008 (primary carcinoma and lymph node metastasis), as well as from snap frozen carcinoma and lymph node metastasis. DNAs were screened for HPV by PCR and subsequent sequencing analysis and were all positive for HPV6. Co-infection with 37 other low- and high-risk HPVs was excluded by multiplex hybridization of group-specific PCR products with HPV-type-specific probes.

**Figure 1:**
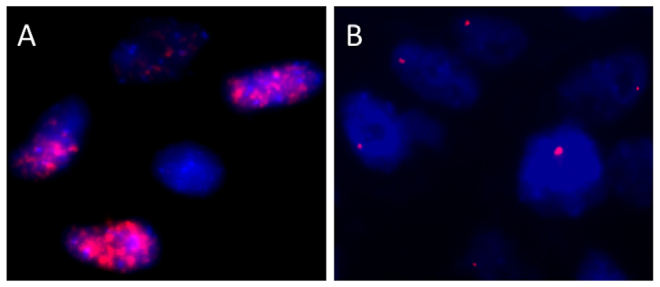
FISH analysis of tissue sections from the reported patient. Probes were directed against HPV6. Magnification ×750. (**A**) Papilloma. (**B**) Carcinoma.

FISH analysis on paraffin embedded tissue sections confirmed the presence of HPV6 in the papillomatosis (1989) and carcinoma, and furthermore showed diffuse nuclear staining indicating episomal HPV6 in the papilloma (fig. 1A) and nuclei harboring a single punctate signal indicating HPV integration in the carcinoma (fig. 1B). In contrast to HPV16-positive OSCC, however, the laryngeal lesions were immunonegative for p16^INK4A^ expression, whereas p53 showed no detectable expression and pRb was highly expressed (fig. S1 A, D, G).

**Figure 2:**
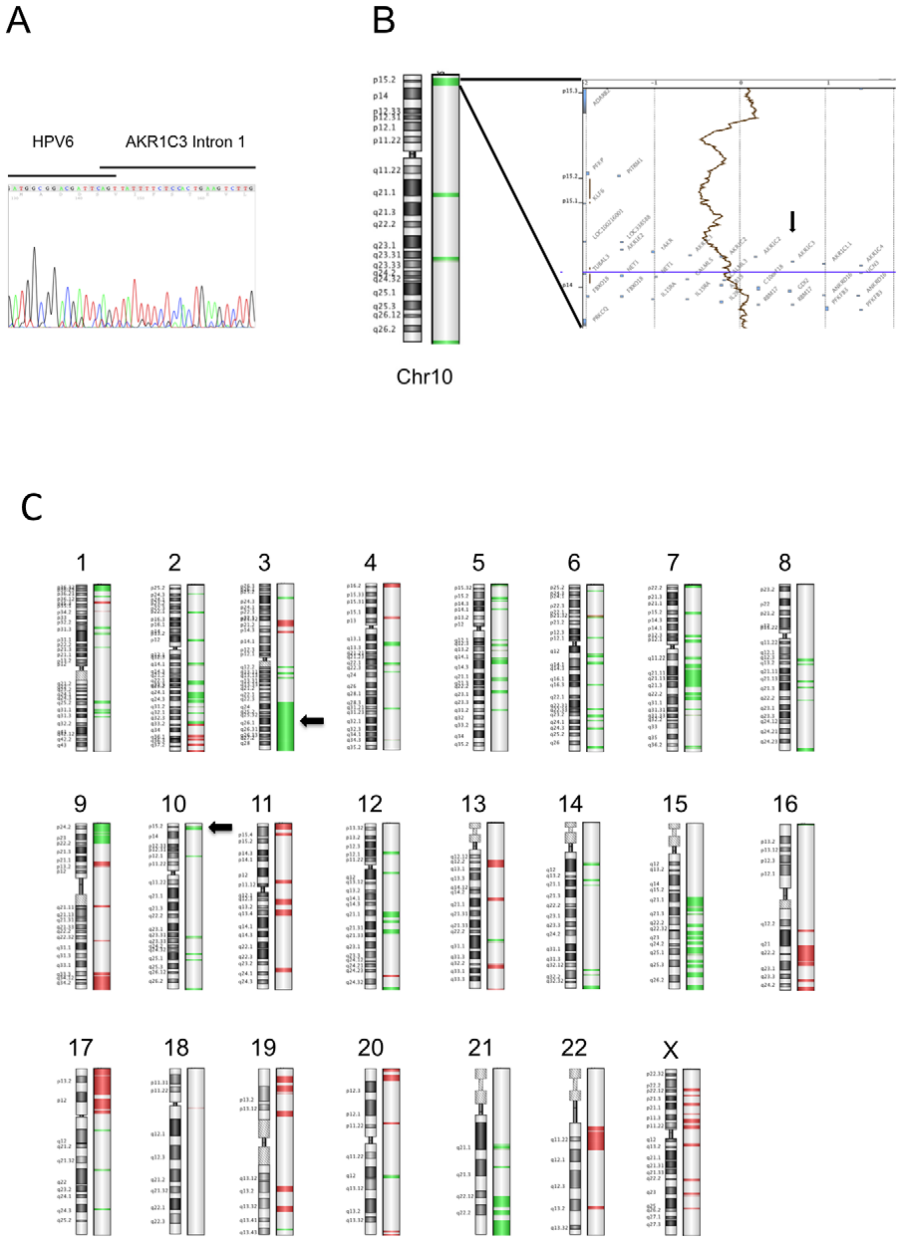
Genetic analysis of the tumor. (**A**) Sequence analysis of APOT PCR product. Analysis of the chimeric HPV/AKR1C3 mRNA showed that it is spliced from the HPV6 splice donor site at nucleotide 745 to intron one of the AKR1C3 gene. (**B, C**) ArrayCGH analysis. (**B**) Large scale ideogram of chromosome 10. AKR1C3 is marked by an arrow. (**C**) Overwiev of all chromosomes. Arrows indicate 3q loss and AKR1C3 integration site on chromosome 10 and a large deletion on chromosome 3. Green colored regions indicate DNA loss. Algorism z-score, Threshold 2.5.

To unmask the location and breakpoint of the HPV6 integration site, we performed a 3′-RACE PCR APOT assay adapted for HPV6 detection using extracted RNA from the frozen carcinoma tissue. Separation of PCR products by agarose gel electrophoresis showed bands indicating viral integration. Subsequent sequence analysis identified integration of HPV6 in the AKR1C3 gene on chromosome 10p15.1 (see fig. 2A). Analysis of the chimeric HPV/AKR1C3 mRNA showed that it is spliced from HPV6 at nucleotide 745 in the viral E1 gene to intron one of the AKR1C3 gene. ArrayCGH analysis using genomic DNA from snap frozen tissue revealed that a 0.57 MB region (10p14–10p15.2) including the AKR1C3 gene was lost in the carcinoma (fig. 2B), thus suggesting deletion of the other AKR1C3 gene copy. ArrayCGH detected additional DNA copy number changes, including a large deletion on chromosome 3 in a region which is normally gained in OSCC (see for the entire CGH profile fig. 2C).

**Figure 3:**
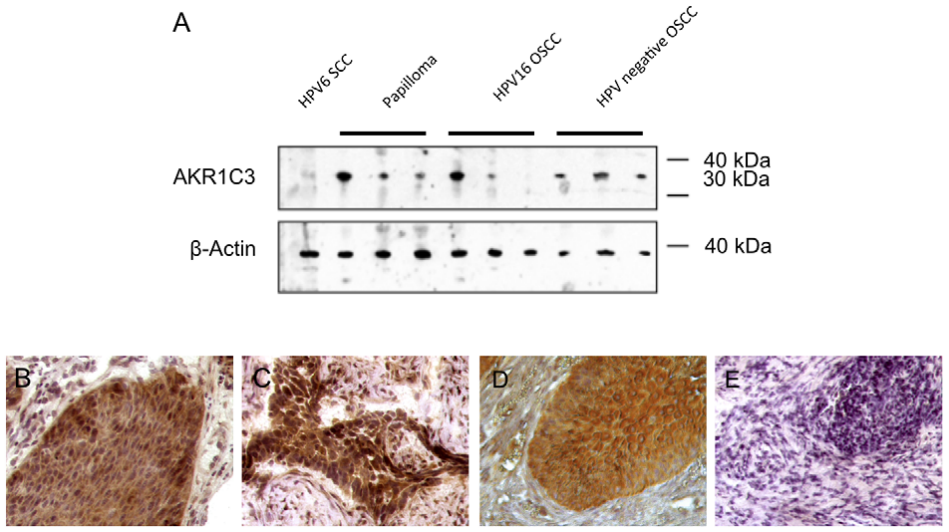
Analysis of AKR1C3 expression. (**A**) Western blot analysis of total protein extracts from dissected tissue samples as indicated. β-Actin was used as an internal loading control (lower panel). Note the faint band of the HPV6 positive SSC while all other protein samples except for one HPV16-positive OSCC showed moderate-strong AKR1C3 expression. (**B–E**) Immunohistochemistry for AKR1C3 expression showing (**B**) strong immunostaining in a control HPV11-positive papilloma, (**C**) strong immunostaining in a control HPV16-positive OSCC, (**D**) strong immunostaining in the papilloma from 1989, (**E**) and no staining in the primary carcinoma from 2008. Magnification ×400.

Western blotting showed a distinct AKR1C3 protein expression in all control samples except for one HPV16-positive OSCC and the laryngeal carcinoma of the here reported patient (fig. 3A). Immunohistochemical staining for AKR1C3 protein expression confirmed the observed expression by western blotting of the controls and the carcinoma. In contrast to the carcinoma, the papilloma showed strong AKR1C3-expression (fig. 3D and 3E).

## Discussion

HPV6 and/or HPV11 have been reported to be present in 50–100% of RRP cases, depending on the methods used for viral detection (7). While the incidence for malignant transformation into squamous cell carcinoma (SSC) in adult-onset RRP varies from 3 to 6% in the literature [31], [32], this is a very rare event in juvenile-onset RRP with a reported transformation rate of less then 1% [33]. Although both low risk HPV types 6 and 11 are capable to cause malignant transformation in adult-onset RRP, HPV11 appears to have the highest potential [34]. HPV11 has also been detected in the 20 reported cases of juvenile-onset RRP that show malignant transformation [6]–[9]. In this study we present the first case showing integration of HPV6 in the AKR1C3 gene on chromosome 10p15.1 in association with loss of its gene expression and carcinoma formation.

Several studies have provided evidence, that integration of high risk HPV types, such as HPV16 and HPV18, is a pivotal step in the transition of cervical intraepithelial neoplasia (CIN) to invasive carcinoma [27], [35]. Also in OSCC, HPV16 is often found integrated [17], [26], [36], [37]. In contrast, the low risk HPV types 6 and 11 are mostly present in the episomal form, both in uterine benign and CIN-lesions as well as in laryngeal papilloma [34], [38]. For tonsillar carcinoma, integration of HPV6 DNA into chromosome 10q24 has been described by cloning of the virus-cellular integration site [39]. In the here presented RRP case, we provide evidence that similar to high risk HPV, also low risk HPV type 6 can integrate into the cellular genome, leading to the production of a virus-cellular fusion transcript by PCR with the cellular sequence derived from 10p15.1. In addition, arrayCGH identified a loss of this sequence as part of a longer deletion of 10p14-p15.2. This is in agreement with other studies showing that HPV-integration can lead in up to 77% of cases to alterations of genomic structures through the amplification, deletion and complex rearrangement of flanking cellular DNA [40]–[42]. However, the mechanism leading to these alterations upon integration is still unclear and remains to be studied. Because Wentzensen et al. [35] reported that high risk HPV DNA can be integrated at sites located all over the host genome with a general predilection for fragile sites, we performed a database query for fragile sites at 10p. No listed fragile sites were identified near the HPV6 integration site [43].

The cellular part of the identified fusion transcript consisted of intron one DNA of the AKR1C3 gene, which codes for a hydroxysteroid dehydrogenase involved in the regulation of local concentrations of androgens and estrogens particularly in hormone dependent tissues like prostate, breast and endometrium. Cancers originating from these tissues are associated with an upregulated protein expression [44]. In a most recent article Wanichwatanadecha et al. show that AKR1C1 and AKR1C3 protein levels are upregulated under the influence of HPV16 E6-protein and are modulated by truncated 16E6*I protein in cervical cancer cell lines, implicating a distinct role of the AKR1Cs in HPV-related carcinogenesis [45]. Our western blot and immunohistochemical results show that the AKR1C3 protein is also highly expressed in the laryngeal papilloma, but is downregulated in the carcinoma. Because we found a deletion of the 10p14-p15.2 region by aCGH, it is tempting to speculate that one copy of the AKR1C3 gene is truncated by the virus and the other copy is lost during transition to carcinoma. In this deleted chromosomal region, however, also close homologues of AKR1C3 are located, including AKR1C1, AKR1C2 and AKR1C4. These AKR1C family members are also involved in steroid metabolism [44], and selective loss of AKR1C2 in prostate cancer, for example, have been found to promote clonal expansion of tumor cells by enhancement of androgen-dependent cellular proliferation [46]. More studies are thus required to further elucidate the exact roles of the AKR1C members in HPV-related cancer.

We noticed that p16^INK4A^ immunohistochemical staining was negative in the here presented RRP case. This is in line with findings of Sano et al. [47], who showed only focal and weak p16^INK4A^ staining in HPV6-positive cervical and genital lesions. Also a pilot study on premalignant head and neck lesions was in agreement with this finding [48]. An explanation for this observation might be differences in the interaction of E6 and E7 oncoproteins with cellular proteins between low- and high-risk HPVs, leading only to p16^INK4A^ upregulation in the case of high risk HPV-related carcinomas [49]. P16^INK4A^ expression thus appears to be an unreliable surrogate marker for low risk HPV-positive head and neck cancers.

Immunohistochemical staining showed no detectable expression of p53 and high pRb expression in the carcinoma. These findings are in line with a report by Arany et al. (1993), also showing low p53 expression and strong pRb expression in skin lesions infected by HPV6. The latter finding might be explained by the inefficiency of HPV6 E7 protein to inactivate pRb [50], which is in contrast to HPV16 positive OSCC in which E7 effectively can downregulate this target [51]. The situation for p53 is more complicated, because p53 showed to be wild type in the carcinoma and no p53 deletion was detectable by arrayCGH. Nevertheless, p53 expression could not be detected by immunohistochemistry. An explanation for this finding could be transcriptional deregulation or deregulation by high MDM2 levels.

In conclusion, this is the first study reporting malignant transformation of juvenile-onset RRP associated with HPV6 infection. Our analysis showed viral integration in the AKR1C3 gene on chromosome 10p15.1 in association with deletion of the chromosomal region 10p14-p15.2, transcription of a virus-human fusion product as well as loss of AKR1C3 protein expression. A more general role for deregulated expression of AKR1C3 and its family members in HPV-associated tumors remains to be studied.

## Supporting Information

Figure S1 **Routine immunohistochemical analysis of known HPV-related proteins.** (**A–C**) Immunohistochemistry for p16^INK4A^ expression showing (**A**) no expression in the primary carcinoma from 2008, (**B**) strong immunostaining in a control HPV16-positive OSCC and (**D**) no immunostaining in a control HPV16-negative OSCC. (**D–F**) Immunohistochemistry for p53 expression showing (**D**) no expression in the primary carcinoma from 2008 in comparison to positive normal epithelium in the same sample (shown in box), (**E**) no immunostaining in a control HPV16-positive OSCC and (**F**) strong nuclear immunostaining in a control HPV16-negative OSCC. (**G–I**) Immunohistochemistry for pRb expression showing (**G**) cytoplasmic and nuclear expression in the primary carcinoma from 2008 in comparison to positive normal epithelium in the same sample (shown in box), (**H**) no immunostaining in a control HPV16-positive OSCC and (**I**) cytoplasmatic and nuclear immunostaining in a control HPV16-negative OSCC. Magnification ×400.
(TIF)
