# Supplementary figures and images for: Integration of HPV6 and Downregulation of AKR1C3 Expression Mark Malignant Transformation in a Patient with Juvenile-Onset Laryngeal Papillomatosis
Source: PLoS One. 2013 Feb 20;8(2):e57207. doi: 10.1371/journal.pone.0057207 (PMC3577740; doi:10.1371/journal.pone.0057207)

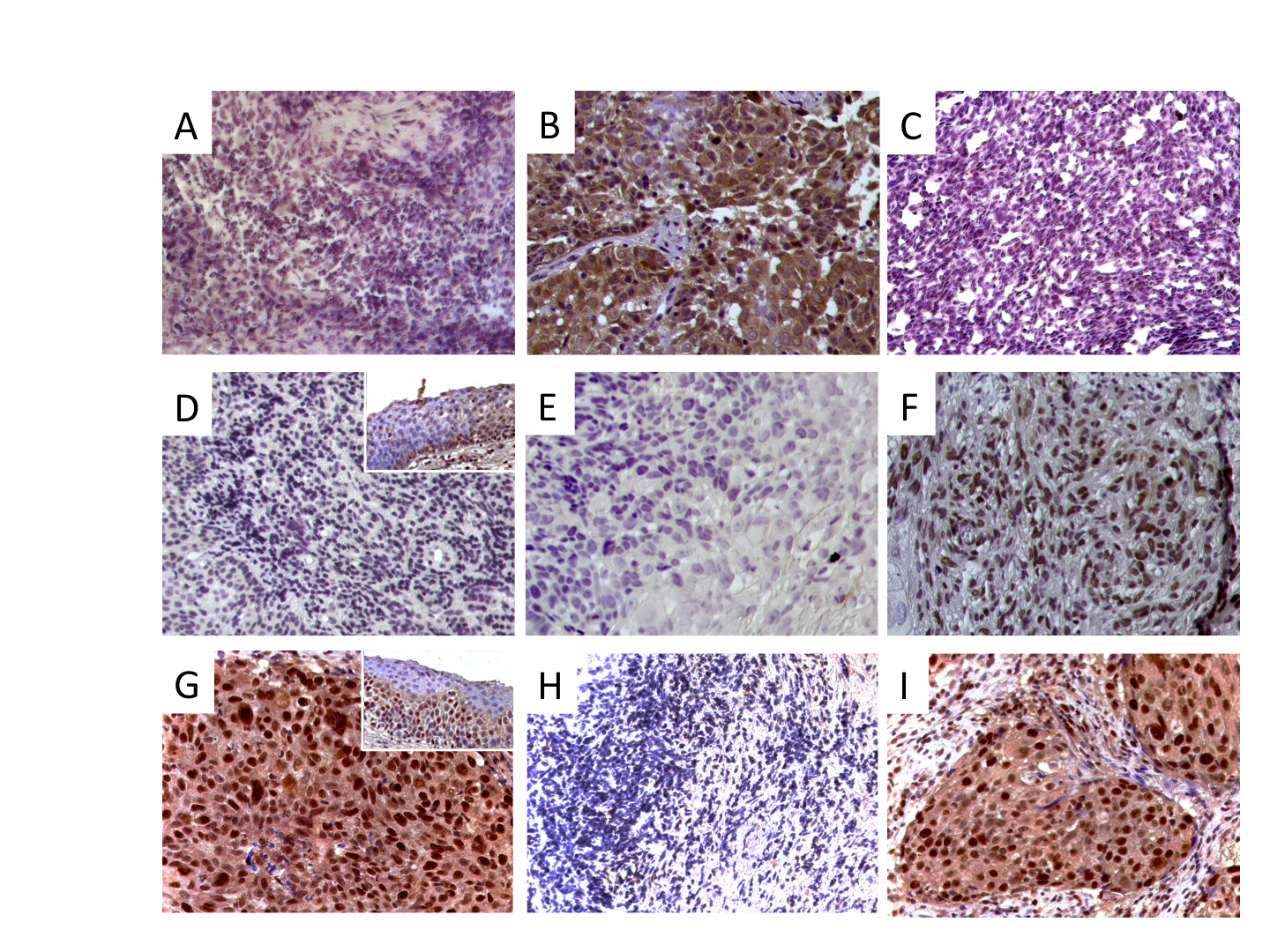

Supplement: Figure S1 — Routine immunohistochemical analysis of known HPV-related proteins. (A–C) Immunohistochemistry for p16INK4A expression showing (A) no expression in the primary carcinoma from 2008, (B) strong immunostaining in a control HPV16-positive OSCC and (D) no immunostaining in a control HPV16-negative OSCC. (D–F) Immunohistochemistry for p53 expression showing (D) no expression in the primary carcinoma from 2008 in comparison to positive normal epithelium in the same sample (shown in box), (E) no immunostaining in a control HPV16-positive OSCC and (F) strong nuclear immunostaining in a control HPV16-negative OSCC. (G–I) Immunohistochemistry for pRb expression showing (G) cytoplasmic and nuclear expression in the primary carcinoma from 2008 in comparison to positive normal epithelium in the same sample (shown in box), (H) no immunostaining in a control HPV16-positive OSCC and (I) cytoplasmatic and nuclear immunostaining in a control HPV16-negative OSCC. Magnification ×400. (TIF) [file pone.0057207.s001.tif]
